# A Multilevel Isolation Forrest and Convolutional Neural Network Algorithm for Impact Characterization on Composite Structures

**DOI:** 10.3390/s20205896

**Published:** 2020-10-19

**Authors:** Amin Ebrahim Salehzadeh Nobari, M.H.Ferri Aliabadi

**Affiliations:** Department of Aeronautics, Imperial College London, Exhibition Road, South Kensington, London SW7 2AZ, UK

**Keywords:** Piezo-Electric sensors, Convolutional Neural Networks, minimalistic and automated, feature extraction, Isolation Forests

## Abstract

In this paper, a Deep Learning approach is proposed to classify impact data based on the type of impact (Hard or Soft Impacts), via obtaining voltage signals from Piezo-Electric sensors, mounted on a composite panel. The data is processed further to be classified based on their energy, location and material. Minimalistic and Automated feature extraction and selection is achieved via a deep learning algorithm. Convolutional Neural Networks (CNN) are employed to extract and select important features from the voltage data. Once features are selected the impacts, are classified based on either, Hard Impacts (simulated from steel impactors in a lab setting), Soft Impacts (simulated from silicon impactors in a lab setting) and their corresponding location and energy levels. Furthermore, in order to use the right data for training they are obtained from the signals as anomalies via Isolation Forests (IF) to speed up the process. Using this approach Hard and Soft Impacts, their corresponding locations and respective energies are identified with high accuracy.

## 1. Introduction

Composite structures face many environmental challenges, e.g., debris impact in aircraft, which can lead to damage such as delamination, compromising the integrity of the structure. With Structural health monitoring (SHM) real time data can be produced and used leading to information on the status of the monitored structure. Application of composite materials have become mainstream in aerospace engineering due to their superior material properties. The Boeing 787 and Airbus 350 XWB utilize composite materials to up to and more than 50% of their volume, and the percentage is still rising [1,2]. SHM is receiving attention as a possible replacement to routine non-destructive inspection (NDI) for maintenance. This is due to the fact that various types of failure and damage can occur from low velocity impacts during maintenance or debris impact from takeoff and landing, that needs to be detected before they lead to failure. These can be indentation, delamination and cracking [3,4]. Different nondestructive inspection methods used included: eddy current, optical, ultrasonic inspection, vibration-based analysis, ultrasonic guided wave for detecting impact damage [5,6,7,8].

Depending on the type of the Transducers, SHM can be divided in two categories of Passive and Active. For active both actuator and sensors are necessary. The Monitored structure is excited by the actuator and the sensor picks up the response. The changes in signals can be used to identify the damage severity and location [9]. For Passive on the other hand, only sensors are required, where they continually monitor the structure. In this case the system needs to generate a specific actuating signal [10]. Transducer layout and configuration on the monitored structure can be optimized to increase detection accuracy, while avoiding unacceptable additional weight and costs [1,11,12]. Printed circuit boards (PCB) have been developed that activate at the indication of an impact [1]. This device can be used to collect impact data from piezoelectric sensors located on the body of aircraft. After data is collected, post processing in the data is crucial. After the data is cleaned up via digital filtering algorithms needs to be processed to detect Impacts within the composite structure. Interpretation of SHM data is done via measured parameters that are trained with machine-learning (ML) techniques [13]. Various techniques have been proposed such as Image processing techniques [14] using filtering and enhancement techniques to denoise the image and perform alignment and artefact correction [15]. Worden and Mason have illuminated the utility of machine learning to damage identification concluding that neural networks are still popular, and systems like support vector machines are beginning to appear more regularly [16,17,18].

## 2. Background

As a crucial step of any ML algorithm, data mining is the process of detecting patterns within data. The detected patterns will be used to predict and help with detecting outliers and hence decision making, while representing the patterns in terms of structures, facilitate the extraction of conclusions on the patterns. [16]. Machine learning techniques can be classified into three broad categories according to the nature of learning: (1) supervised learning, (2) unsupervised learning, and (3) semi-supervised learning [19]. Supervised learning provides a learning scheme with “labelled data”, i.e., examples that include specified outputs (pairs of input data and output data). Using labelled data, rules are developed to classify new data sets. Unsupervised learning encompasses the detection of patterns within the data sets consisting of “unlabeled data”, i.e., data sets with unspecified outputs, which fit to a general rule and can, therefore, be grouped together. Unsupervised learning is highly suitable in SHM applications for detecting unlabeled data as labeled data would be very expensive to come by, e.g., while supervised learning can be utilized to detect the type and severity of damage [20]. Semi supervised learning, representing a combination of the two learning schemes, typically aims at obtaining a classification of data using both labelled and unlabeled data. Semi supervised learning schemes have been applied, combined with other monitoring techniques to extract information on modal characteristics of bridges [16,21]. Unsupervised techniques seem to fit the criteria of determining impacts and anomalies from a data set. There are various unsupervised learning techniques that can be used to detect certain patterns within a group of data and extract a specific feature from the mentioned data set. Choy [22] has used an index-based approach whereby using Convolutional Neural Networks (CNN) on the Root Mean Square Deviation (RMSD) obtained via Impedance measured from the piezo electric sensors. Different CNN models have been implemented to improve performances especially in Image Processing. The main differences between different models are the number of layers and the interconnection structures [19,22,23]. This method can be a viable method however the RMSD can be easily influenced by errors and variations within testing conditions. Furthermore, the number of layers required within the CNN can also be an issue as determining the correct number of layers can add to computational complexity, and if the algorithm is to be embedded on a chip, the extra added computational complexity can cause further difficulty.

Used for unlabeled data, clustering is a pattern detection technique with many dimensions. Interest in clustering has built among researchers in many fields such as intrusion detection and damage loculation [24]. Clustering’s main advantage is being able to learn from and detect patterns from raw data without explicit labels [25]. This method suggests that normal data will cluster around one another and anomalistic data are far from such clusters. Taking advantage of separability of hyperplanes in higher dimensions One-Class Support Vector Machine (1-SVM) aims to construct a hyperplane decision boundary, where the training points are assumed to belong to one class and all non-training points belong to another class [26]. Taking its roots from isolation tree methods, binary forests such as Isolation Forest (IF) starts by utilizing all nodes to randomly select a dimension and afterwards randomly select a splitting threshold. The algorithm continue until each a single sample is allocated to each node. Via this method, an ensemble of trees is created. Samples with unusual values (or outliers) have a higher chance to be isolated earlier on in the tree growing compared to samples in clusters, therefor the average depth/length of the sample within the trees in the ensemble leads directly to an abnormality score [26].

Based on the results by [26], the 1-SVM and IF method seem to be the most accurate method. IF seems to be more advantageous since it requires less processing time and targets abnormalities right away rather than identifying normal data first then classifying abnormalities. Elliptic Envelope (EV) can be used to fit data to a normal distribution and identify abnormalities. Afterwards IF can be used to find impacts within anomalistic data set, however IF on its own is enough to detect anomalies which will include the impacts. While IF requires the number of trees to be determined by the user, it is needed in order to fully fetch out the anomalies within a data set which will hold the impacts. This will also reduce the need for heavy filtering. However, further steps are needed to classify the impacts and their energies. In order to have an automated process as much as possible, a Deep Learning approach is most beneficial. CNN’s have been used extensively in Image Processing for classification of different image models. The approach in [27] has shown that CNN’s can be sued for impact detection, localization and characterization. The data needed for training the model can be collected via Piezo-Electric sensors mounted on aircraft body (either the wings or the fuselage). One way to improve on the CNN approach for detection of lower energy impacts (but still of significance) is the addition of RNN’s.

## 3. Experimental Setup

**Figure 1:**
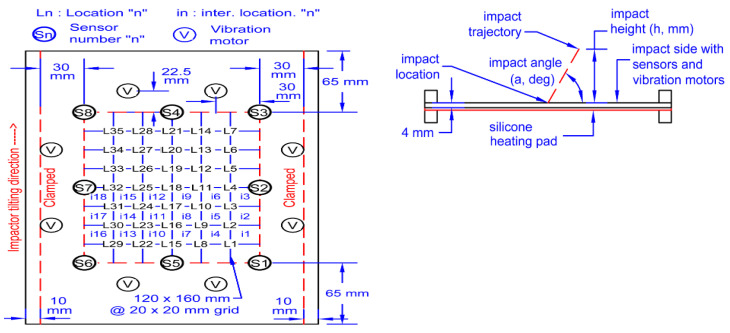
Sensor setup.

**Figure 2:**
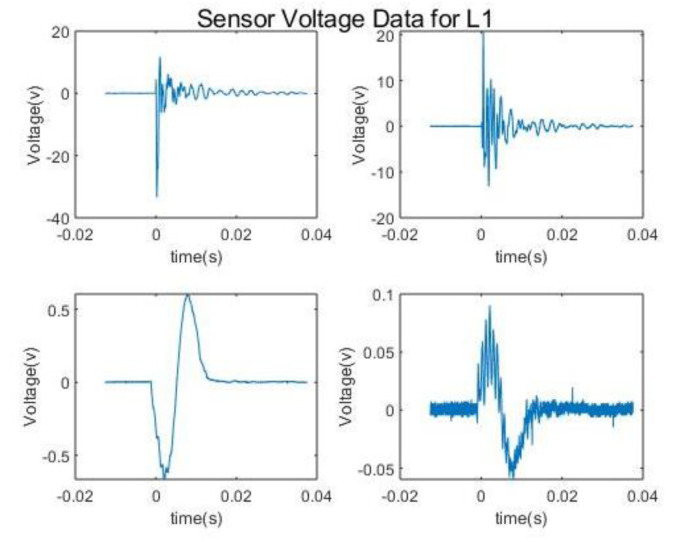
HI and SI for sensor 1 on the right and the HI and SI for sensor 8 on the left.

**Figure 3:**
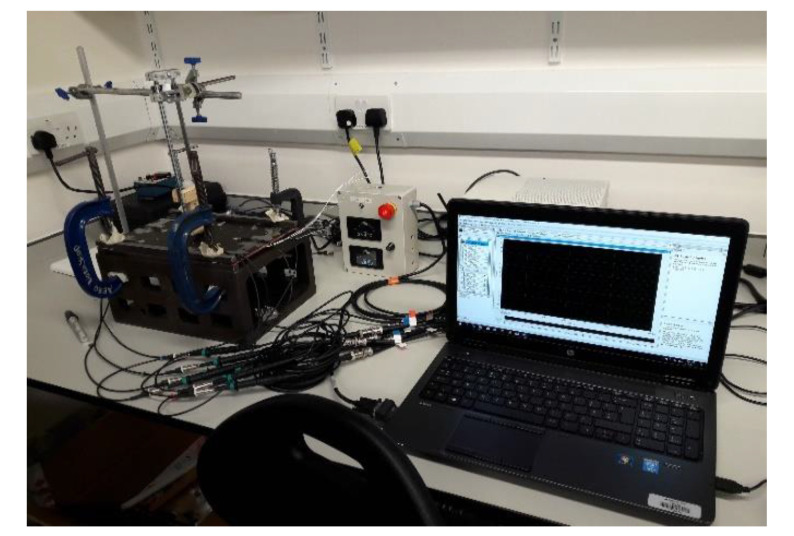
Experimental setup for impact data collection.

The data are collected from a set of Piezo electric sensors mounted on a composite structure. In this case, the structure used is a rectangular carbon fiber plate. Figure 1 shows the positions of the sensors. The sensor boding and quality have been verified via electromagnetic impedance (EMI) analysis. The plate is clamped on both ends while connected to a vibration motor to simulate real life vibrational noise. The shown locations (L1, L2, L3…) are used for impactors to be dropped from various heights on the plate to simulate real life impacts. Two types of impactors are used: Steel impactor for Hard Impacts (HI) and Silicon Impactors for Soft Impacts (SI). The impactors are dropped from heights of 50 and 100 mm. The voltage data is collected with 1 MHz sampling frequency and sent to a computing software (such as lab view) via a D-connector and USB connection to a laptop or PC. There, the data are allocated to each of the corresponding sensors within an Excel File where it can be later used for training the model. Figure 2 and Figure 3 show sample of the voltage data and experimental setup respectively.

## 4. Isolation Forrest

**Figure 4:**
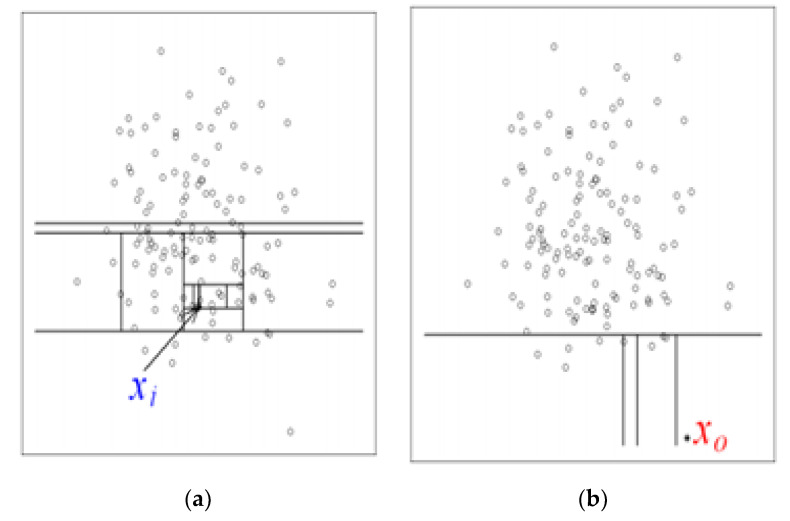
Examples of normal vs. abnormal data: (**a**) indicates how X_i needs more splits and trees to reach the data. (**b**) indicates how X_o, an anomaly, needs much less trees.

**Figure 5:**
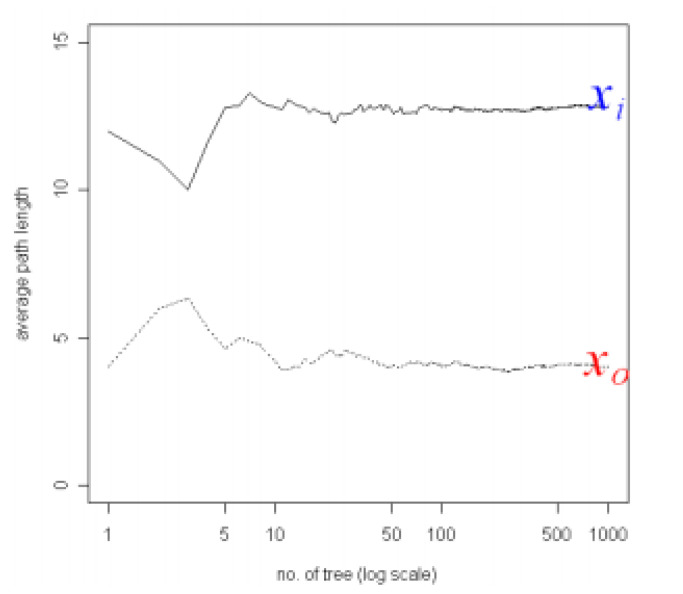
Anomalies require a shorter path length for the algorithm to isolate it compared to a normal data point.

Isolation Forest (IF) is mainly based on the idea of isolation and decision trees, and how the two are utilized for anomaly or outlier detection. The trees, referred to as iTrees are built for a given data set and the anomalies are instances which have the shortest average path length on the iTrees. In principle outliers are much less frequent than the regular data points (also within the feature space), hence why such random portioning, outliers are identified closer to the root of the tree. In iTrees, partitions are created by initially randomly selecting a feature and then selecting a random split value between the min and max values of the selected features. An example of a normal vs. abnormal observation is shown in the Figure 4 and Figure 5 [28].

There are two main variables involved in IF: The number of trees and the subsampling size. The path length of a point is shown as *h(x)*, and is known as the number of edges or paths it takes to reach a certain data point. In order to classify a point as an anomaly, an anomaly scoring method is utilized by IF, as discussed further. A way of measuring the number of nodes it takes to reach a point, should also be taken into consideration. This is shown as the average path length of an unsuccessful search, shown by *c(n)*, where n is the node:

(1) c(n) = 2 H(n−1)−(2(n−1)/n)

where H(n) is ln(n)+0.5772156649. As c(n) is the average of h(x), given n, it can be used to normalize h(x). Hence the anomaly score is obtained via:

(2) $$s\left(x,n\right)\;=\;2^{\frac{-E\left(h\left(x\right)\right)}{c\left(n\right)}}$$

where E(h(x)) is the average of h(x) from a collection of IF’s. Hence:

- Instances where *S* is returned close to 1: The point is an Anomaly.
- Instances where *S* is returned close to 0: The point is Normal.
- Instances where *S* is returned close to 0.5: The point does not have any distinct anomaly.

**Figure 6:**
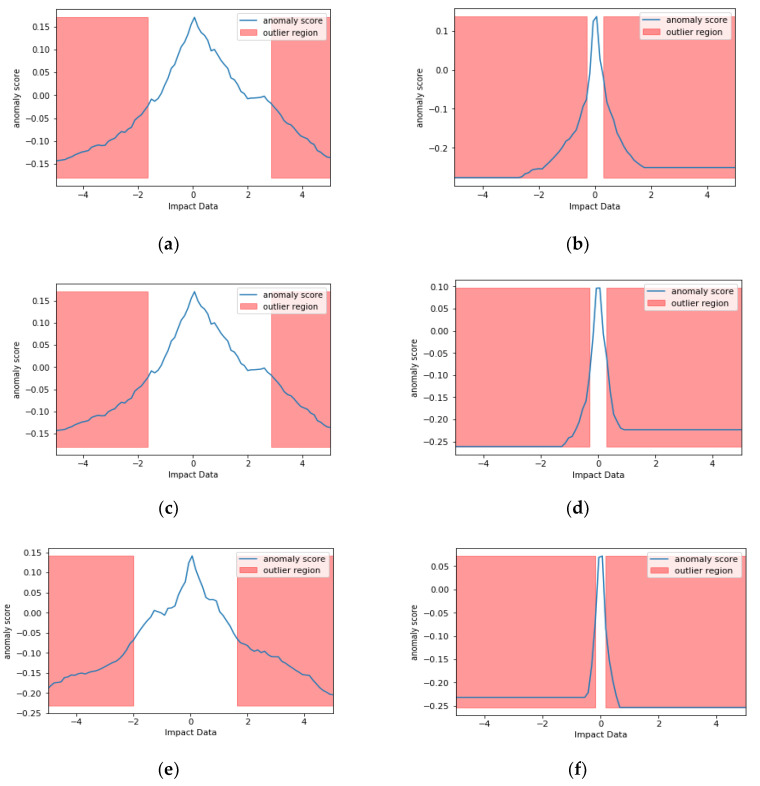

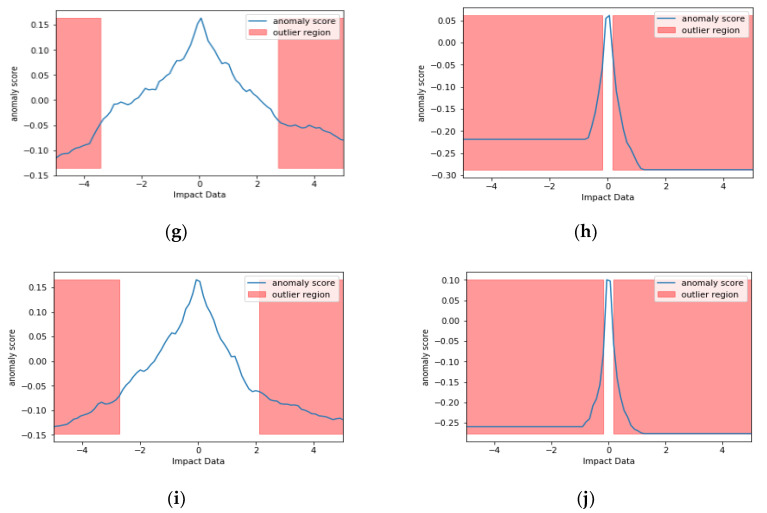
The red area indicates outliers within sensor data as follows: (**a**,**b**) IF analysis for sensor 1; (**c**,**d**) IF analysis for sensor 2; (**e**,**f**) IF analysis for sensor 3; (**g**,**h**) IF analysis for sensor 7; (**i**,**j**) IF analysis for sensor 8. Impact data refers to the voltage data from the sensors. Note the constant anomaly score on the higher voltage impact data on the noisy data.

The Scikit Learn package within Python can be used to do this, however, the anomaly score is offset by −0.5. Usually it should be in the [0,1] range. In the case of the Python package it is offset and in the range of [−0.5, 0.5]. Negative values are outliers, positive values are inliers. Figure 6 shows examples of IF in action on two signals impact signals produced by a hammer. Figures on the left side show the signal without vibrational noise for sensors 1, 2, 3, 7 and 8, and the ones on the right with vibrational noise for the same sensors. 100 trees where used for training with random selection of features.

So far IF has been explained through a mathematical context. In the context of SHM, IF can be highly beneficial as the important voltage data can be extracted with minimal effort from the noise and vibrations during flight. While it might be easy to assume that the algorithm simply targets the peaks of signals, through its sub sampling and random forest approach, it can also extract lower peaks that are situated out of the noise and vibrations.

As it can be seen from the data without noise, near the abnormal points there are certain number of points that have the same score at a range of voltages. The noisy data happen to have a very small set of points that are not abnormalities. Based on Figure 2, this makes sense as there is noise added on top of the impact from the setup, hence more abnormalities, i.e., the background noise adds to the impacts signal in certain points. It should be noted that these abnormalities can contain the impacts.

## 5. Convolutional Neural Network (CNN)

CNN is a deep-learning algorithm that has been extensively researched in the last few years, creating many variations [29]. The natural visual perception mechanism within living creatures was the main motivation behind the algorithm. The first CNN, influenced by the discovery of Kunihiko Fukushima was the LeNet-5, by [30]. Kunihiko Fukushima had found that neurons in the visual cortex are responsible for detecting light in receptive fields [31]. CNN’s are built on multi-class classifications where each image is associated to label within a class, from which the network trains its weights. Many parameters can be represented by a class, including: a point of impact, impact localization, an energy level or any other [27].

The method in this paper utilizes a CNN as the second step for feature extraction after IF to find anomalies from the impact data. From the outputs of PZT sensors, the proposed model takes the raw data as 2D images, for different impact locations and energy levels, therefore outputs are given as the class with the highest probability [27]. Many implementations of the CNN exist, but they all include three layers: convolution, pooling and fully connected layers. The convolution layer consists of many multiple convolution kernels that learn features of the input data via feature extraction and selection and thus generating feature maps. Feature maps are created by initially convolving the input with a learned kernel, and next, by applying on the convolved results an element-wise non-linear activation function. In mathematical terms, the feature value $Z_{i,j,k}^{l}$ at a certain location (i,j) in the *kth* feature map of the *lth* layer, is [27]:

(3) $$z_{i,j,k}^{l}\;=\;W_{k}^{l}x_{i,j}^{l}\;+\;b_{k}^{l},$$

With $W_{k}^{l}$ and $b_{k}^{l}$ representing the weight vector and the bias term respectively of the *kth* filter of the *lth* layer. The kernel $W_{k}^{l}$ is shared with the $Z_{i,j,k}^{l}$ feature maps, differing from typical ANN’s. This is advantageous since it reduces the model complexity hence making it easier to train. With the non-linear activation function called a(·), then the action value $a_{i,j,k}^{l}$ of convolutional feature $Z_{i,j,k}^{l}$, is [27]:

(4) $$a_{i,j,k}^{l}\;=\;a(z_{i,j,k}^{l}),$$

The activation function is crucial since it introduces non-linearities to the CNN, making it robust for detecting non-linear features. Sigmoid, ReLu and tanh are the most used activation functions. The pooling layer between two convolution layers has the role of minimizing the feature maps resolution via shift-invariance. With the pooling function defined as pool(·), then for each feature map [27]:

(5) $$y_{i,j,k}^{l}\;=\;pool\;\left(a_{m,n,k}^{l}\right),\forall \left(m,n\right)\in R_{ij},$$

With $R_{ij}$, a local neighborhood around location (i,j). Pooling operations can involve Average pooling and max pooling are used for pooling operations. Usually, kernels in higher layers obtain more robust features such as energy levels or natural frequencies, while the ones in the lower layers encode more low-level features such as curves or edges. At the end of the network, the fully connected layer connects all the neurons in the previous layer to the ones in the current layer, creating global semantic information. The last layer is the output layer, where a SoftMax operator is employed (or SVM) [27]. This reduces the quantity of network parameters due to improving the efficiency of the forward function. The difference from a typical neural network is that the CNN have neurons arranged in three dimensions: width, height and depth.

The **convolution layer** is used for extracting information from raw input data utilizing multiple automatically taught filters where the data’s features are obtained. The user determines the filters size and numbers. The filters scan the input from the upper left-hand side corner to the bottom right hand side corner, each creating a feature map [27].

The **pooling layer** down-samples the width and height, reducing input dimensions hence, reducing the number of parameters to be computed. This overall reduces overfitting and the complexity of the network. This is done on each depth slice of the input separately, down-sampling them all. Various patches are created by dividing each, equal in area to the filter size set initially by the user when defining the pooling layer [27].

The **flatten layer** turns the input into an array of one neuron depth and height, equal in length by changing the inputs shape to the product between the length, depth and height of the input to that layer. The output layer in every CNN must be a one-dimensional vector, hence this step is crucial [27,32].

The **dropout layer** randomly cuts off fractions of the nodes in the network reducing overfitting. This can be used to replicate a great number of different architectures [27,32].

The **densely connected layer** connects each output neuron to all the neurons from the input. This is implemented at the output along with A SoftMax function to produce predictions along with the output are responsible for implementing this step. Thus, the nodes at the output of the layer contain the probabilities of the input to the CNN belonging to all classes. Each neuron, containing the convolutional and pooling layers, receives all the information from the first half of the network, since they are connected to all the neurons of the input layer [27,33,34].

The instructions to create an optimum architecture of a CNN are not generally clear. The most common way of finding the best structure is via trial and error. A function which truly represents the accuracy and efficiency of the prediction is a fundamental step. One parameter which can quantify the performance of an algorithm for each class, is the Confusion matrix. To show the effectiveness of CNN on an individual HI and SI, the results of the confusion matrices is shown in Table 1 and Table 2

**Figure 7:**
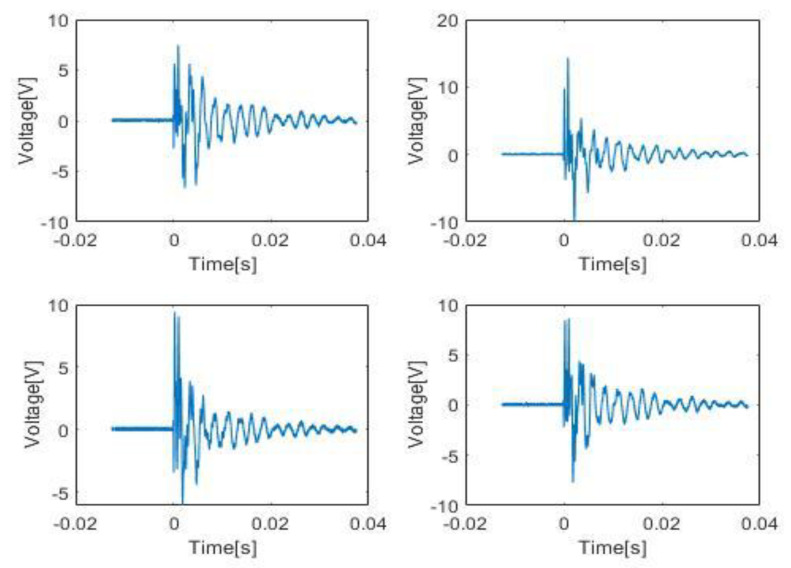
HI’s from four sensors.

**Figure 8:**
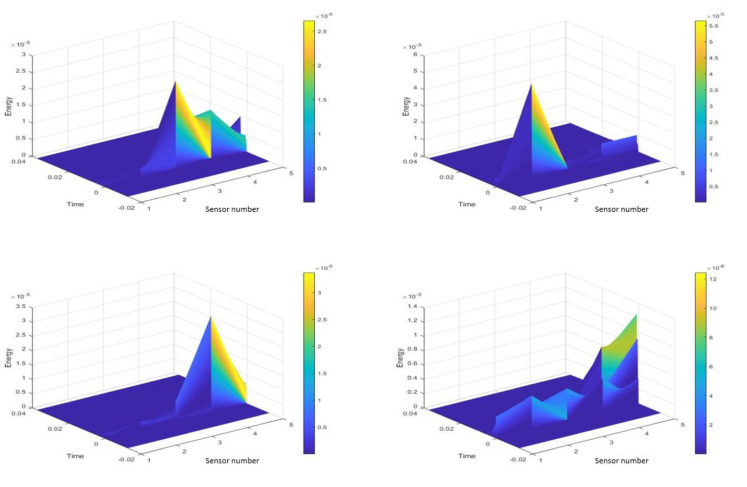
Energy spectrum of sensors 1 to 5 for four different HI’s.

**Figure 9:**
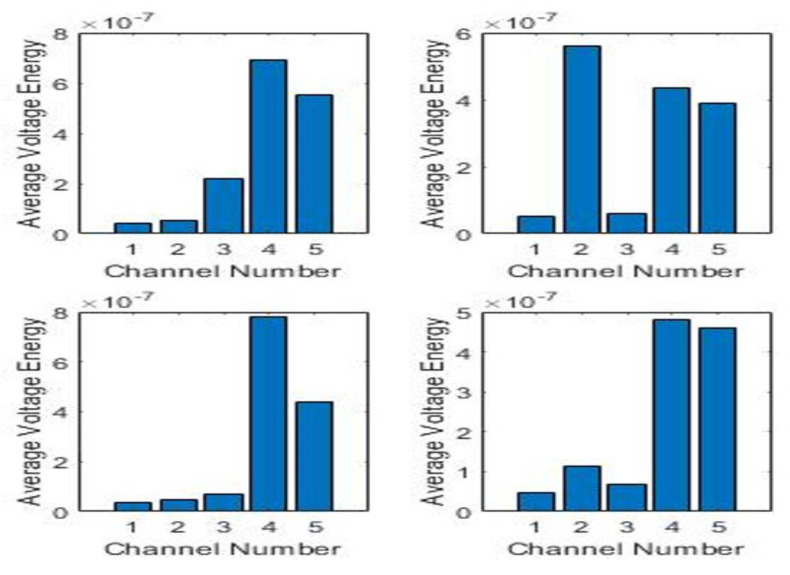
Average energy for sensors 1 to 5.

Looking at the results from Table 1 and Table 2 the CNN algorithm is more than capable of detecting individual impacts by being trained by a set of 4000 individual impacts. For HI and SI each were trained with the same type of Impact. The training is more accurate for impacts that have been dropped from the same height as they all have the same energy. The ones dropped from different heights and using different masses have a lower accuracy as their features are harder to select and extract. Next the results for Energy classification on the individual HI and SI will be shown. In order to tackle Energy classification, initially the Energy of the individual impact must be obtained. There are various methods to do so, however, the best method would be to use the capacitance energy of the PZT sensors obtained by:

(6) $$E=\;\frac{1}{2}C_{PZT}V^{2},$$

where $C_{PZT}=45nf$. Afterwards the average energy is computed for each of the channels via:

(7) $$E=\;\frac{1}{N}\sum_{n=1}^{N}E^{ij},$$

where *j* = 1,2, 3…,8 is the channel number and *N* the total number of samples per sensor, which in this case is 100,000. Aim is to divide the impacts into three energy categories: Low, Medium and High. Training for energy classification would have to take place separately from that of the impacts. Looking at the individual HI for four sensors, as shown in Figure 7, their energy spectrum has been obtained via MATLAB as shown in Figure 8 and their average energy using (7), is shown in Figure 9.

Using the average energy data obtained, the training of the energy CNN can begin. The results of the training and testing for both HI and SI (SI energies also obtained with the same method) are shown in Table 3 and Table 4

This shows that the energy CNN is more than capable of classifying the energy levels of different impacts. For SI, again since the features are less obvious, the algorithm can’t obtain as good results as the HI’s energies.

## 6. Impact Localization

**Figure 10:**
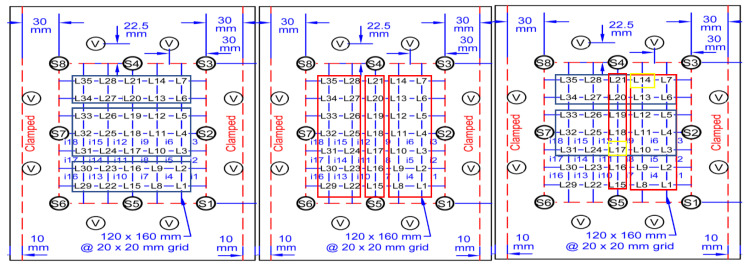
Division process of the plate to different location classes.

This section focuses on impact localization, via another individual CNN after initial IF step. Looking at Figure 10:

The aim of dividing the plate into different divisions is to try initially find the general impact location based on the time of arrival, into three general horizontal location classes. The same is done but by finding the general impact location based on three general vertical location classes. The overlap of the two types of classes pinpoints a much more accurate are of impact which itself includes either two or three locations. From there, the algorithm once more utilizes the time of arrival to find the impact location within either the two or three smaller classes. The results can be shown in Table 5 and Table 6. The impact localization of the individual HI and SI results show that the CNN used for this method can classify the proper impact location.

## 7. Final Model Structure and Results

So far, the two important components of the final model have been discussed for individual single impact HI’s and SI’s. To test the final model for robustness and more realistic data, two sets of signals are constructed:

- Ten segment signals where many HI, SI and vibrational noise are included.
- Ten segment signals where HI and SI are much more isolated and so the signal is more realistic.

**Figure 11:**
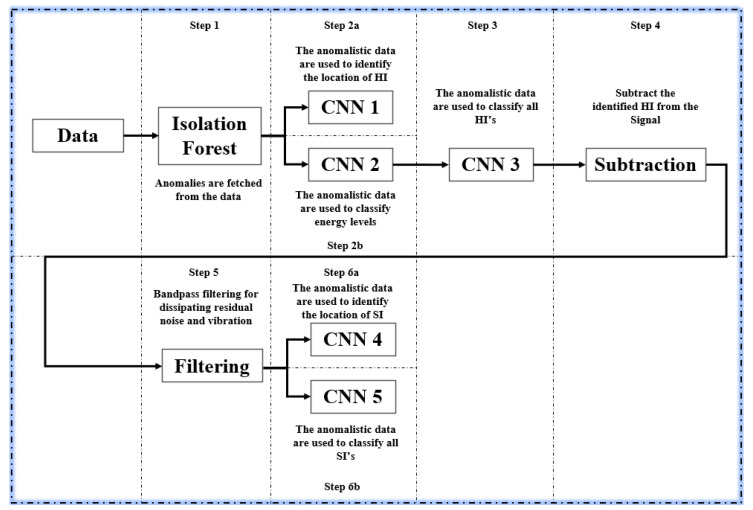
Final model structure.

Signals are obtained by using a function to fetch signals from various impact files and stitching them up to one another. The final model can be summarized in the following steps and Figure 11:

(1) The data are passed into an IF algorithm to have their anomalies fetched and moved on to the deep learning segments.
(2) The anomalies follow two branches:
  (a) One branch with CNN-1 where the impact localization takes place for HI. HI impact locations can be found from this step since HI’s are easily visible to the IF, since as seen in Figure 2, compared to SI, they have higher amplitudes.
  (b) One branch with CNN-2 where the energy classification of the impacts take place.
(3) Following CNN-2, the same anomaly data are passed into CNN-3 where the CNN is specified to look only for HI.
(4) Once HI are found they are subtracted from the anomaly data, leaving only SI and left-over vibrations from step 1.
(5) The remaining data is passed through a band pass filter of cut-off frequencies 100 Hz and 300 Hz (characteristic frequencies for vibrational noise) for dissipating any residual noise and vibration.
(6) The remaining data passed onto:
  (a) CNN-4 where the SI localization is done
  (b) CNN-5 where the SI are identified.

**Figure 12:**
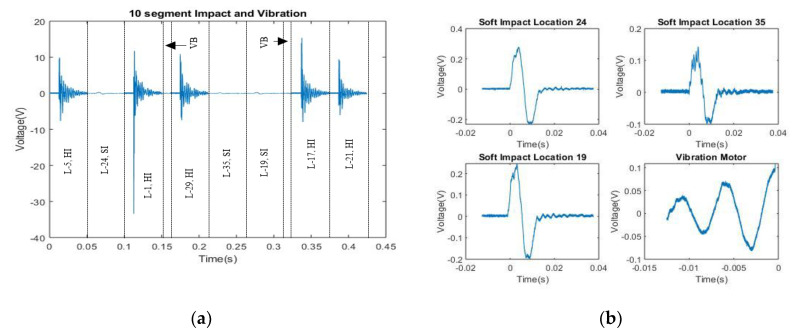
(**a**) Examples of ten segment signal. VB indicates vibration; (**b**) shows isolated SI’s.

**Figure 13:**
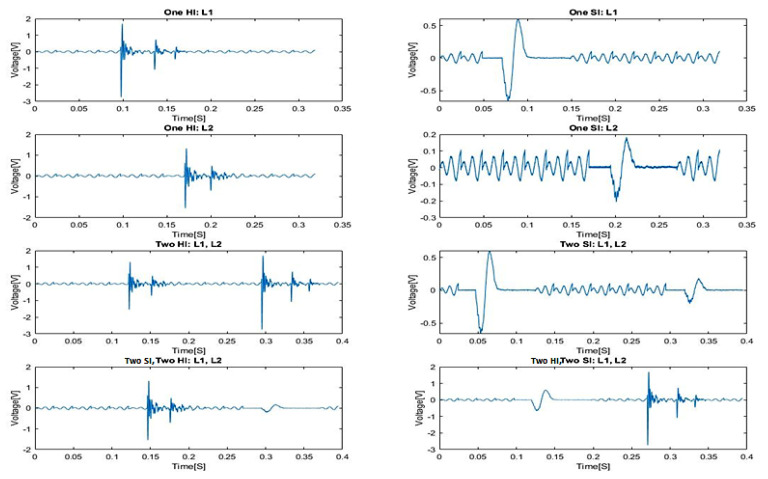
Examples of isolated impacts representing more realistic signals.

Figure 12a shows an example signal containing multiple SI’s, HI’s and vibrations, while Figure 12b shows isolated SI’s. Figure 13 shows a collection of isolated realistic ten segment signals.

**Figure 14:**
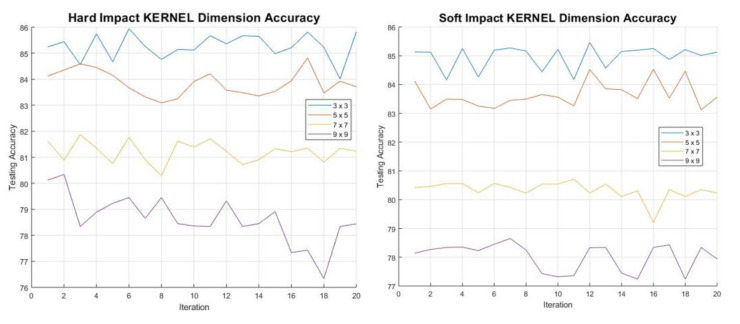

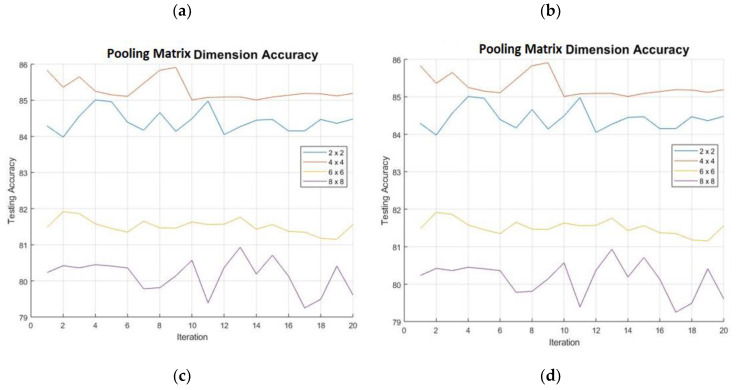
(**a**,**b**) indicate percentage accuracy of HI Kernel and Pooling dimensions respectively; (**c**,**d**) indicate percentage accuracy of SI Kernel and Pooling dimensions respectively.

A total of 1000 data sets have been used for training the model. What needs to be determined is the dimensions of the kernel and pooling matrix. Initially the optimum kernel dimension is determined by looking at each impact type (SI and HI) separately, while keeping the pooling matrix at a minimum of 2 × 2. Figure 13 shows that the optimum dimension for detection of both HI and SI is at 3 × 3. The higher the dimension the less the accuracy. This can be expected as the kernel of larger dimension might miss certain features, leading to lower accuracy. This can be thought of in terms of a moving average. In order to find the optimum pooling matrix, dimension the kernel used to obtain the highest accuracy has been used. The pooling matrix essentially works as a compression matrix. It can be seen in Figure 14, that the 4 × 4 dimension gives the highest accuracy alongside the 3 × 3 kernel dimension. Since the optimum dimensions of both the kernel and pooling matrix have been determined, the model can now be trained. The main objective is for the model to determine the HI and SI with high accuracy. The table below shows the percentage accuracy and computation time for both the HI and SI. It is important to acknowledge that the data used for training and testing are picked at random from different locations and impactors. What is more significant is the height of the impactors which corresponds to the energy levels of the impacts. This has been added to the testing since it is suspected that combining impacts of different energies can affect the training.

The results for the initial sets of signals (10-segment multiple HI, SI, vibrations) is shown in Table 7. The results of the secondary set of signals (10-segment isolated HI, SI realistic signals) is shown in Table 8. As seen from both tables the algorithm can detect HI’s at higher accuracy compared to SI’s. This is since SI’s have less distinct features compared to HI’s and so are harder to detect. The same stands for the energy levels. This method was also compared to other methods previously considered and the comparison is shown in Table 9.

In Recurrent learning (RL), the user must predefine a certain set of features where the algorithm cycles through and picks the ones that give better results after each stage of each cycle. Over time the algorithm converges to a certain percentage accuracy via concatenating the feature set. This method needs the user to specify certain number of features and hence is not viable for the desired function. Long Term Short Memory (LTSM) are a variant of Recurrent Neural Networks (RNN), where special units are used for memory maintenance between each stage of the algorithm. RNN’s themselves are a class of Artificial Neural Networks where the connections between the network nodes create a directed graph along a temporal sequence. This allows the network to exhibit temporal dynamic behavior. Unlike the conventional feed forward neural networks, RNN’s can use their memory to process and model variable length sequences of inputs [35,36,37].

## 8. Conclusions

From the results obtained and shown in Table 6, Table 7 and Table 8, for impacts at both the same and different heights, the HI are detected at higher accuracy compared to SI. This can be expected since HI have more distinct features compared to SI. One specific feature is the very sharp amplitudes produced by HI as seen in Figure 2. This also results in short amplitude time and higher differential compared to SI. As for SI, since their features are less distinct the percentage accuracy is not as high. Another point of note is that the percentage accuracy for the proper energy band gap also follows that of the impacts. This can be attributed to the fact that when different energy levels are present within the same impact the features of impacts with lower energies are overshadowed by those of the higher ones even in the case of the HI.

This can still be used as an indicator of different energy levels and can be beneficial for identifying the energy level of the different impacts. This can then lead to classifying the impacts, ranking them from negligible to highly dangerous with the model. This has further led to insufficient computing power in some cases. This can be highly important since the final version of the algorithm has to be able to perform on PCB. If the model is not optimized to the specifications of certain electronic circuits, it will be severely limited in its operation.

## Figures and Tables

**Table 1:** Results of CNN on the individual HI and SI from the same height.

Impactor Type (IT)	Percentage Accuracy	Computation Time (Min:Sec)	Computing Power (Gb)
HI	98.63	46:34	15.8
SI	90.24	51:47	15.9

**Table 2:** Results of CNN on the individual HI and SI from different heights.

IT	Percentage Accuracy	Computation Time (Min:Sec)	Computing Power (Gb)
HI	92.12	62:31	15.9
SI	83.73	70:24	15.9

**Table 3:** Accuracy of energy classification of HI’s.

Num of Samples	High Energy	Medium Energy	Low Energy
2000	97.23	94.56	93.19
4000	98.78	95.47	94.67
6000	98.76	95.87	94.23
Energy Level	6.45 × 10^−7^ and above	4.23 × 10^−7^~6.44 × 10^−7^	4.22 × 10^−7^ and lower

**Table 4:** Accuracy of energy classification of SI’s.

Num of Samples	High Energy	Medium Energy	Low Energy
2000	90.12	90.34	89.29
4000	90.89	89.92	89.31
6000	92.74	88.34	87.34
Energy Level	3.23 × 10^−7^ and above	1.98 × 10^−7^ ~ 3.22 × 10^−7^	1.97 × 10^−7^ and lower

**Table 5:** Initially the general impact location is found based on the 3 and then total 6 divisions from the horizontal and vertical divisions in slide 3. Based on the location, either 2, 3 or 4 locations are left. It is sensible that the higher the number of classes the harder to pinpoint the location.

Number of Datasets	Location Class	Accuracy (%)
2000	6 (3 then 3)	92.7
4000	6 (3 then 3)	95.4
6000	6 (3 then 3)	96.1
8000	6 (3 then 3)	95.2

**Table 6:** Results of the dataset with the optimal number of samples: 6000.

Number of Datasets	Location Class	Accuracy
6000	2	91.6
6000	3	87.6
6000	4	82.6

**Table 7:** Shows the results of the initial set of signals.

Number of Samples	High Energy	Medium Energy	Low Energy	HI	SI
2000	92.34	91.19	81.29	92.57	71.11
4000	92.41	91.89	81.33	92.61	71.43
6000	92.72	91.36	81.41	92.49	71.32
8000	92.17	91.12	81.28	92.83	71.17
	**Energy Range (mJ)**				
	4.68~8.81	3.33~4.67	0~3.32		

**Table 8:** Shows the results of the secondary set of signals.

Number of Samples	High Energy	Medium Energy	Low Energy	HI	SI
2000	97.87	96.43	89.43	98.47	78.01
4000	97.35	96.86	89.45	98.97	78.74
6000	97.23	96.37	89.91	98.34	78.29
8000	97.84	96.23	89.87	98.67	78.38
	**Energy Range (mJ)**				
	4.82~8.78	3.05~4.81	0~3.04		

**Table 9:** Shows the results of different algorithms.

	**Percentage Accuracy**						
**IT**	**RL**	**LTSM**	**CNN**	**RNN**	**RL-CNN**	**LTSM-CNN**	**RNN-CNN**
HI	80.2	82.67	85.76	82.86	82.11	85.59	88.36
SI	72.4	81.23	85.15	81.84	79.87	84.92	87.47
	**Computation Time (Min:Sec)**						
	**RL**	**LTSM**	**CNN**	**RNN**	**RL-CNN**	**LTSM-CNN**	**RNN-CNN**
HI	16:3	39:5	47:6	43:5	55:3	57:3	68:4
SI	18:4	39:3	47:9	43:6	54:4	58:4	68.6
